# How (Not) to Break Up: Constituent Power and Alternative Pathways to Scottish Independence

**DOI:** 10.1093/ojls/gqad022

**Published:** 2023-10-31

**Authors:** Raffael N Fasel, Shona Wilson Stark

**Keywords:** Scottish independence, Indyref 2, referenda, constituent power, devolution

## Abstract

In October 2022, the UK Supreme Court unanimously held that the Scottish Parliament lacks the power to legislate for a second referendum on Scottish independence (Indyref 2) absent an enabling Order by the UK government under section 30 of the Scotland Act 1998. With no such Order forthcoming, alternative pathways to Indyref 2 are being investigated. In this article, we examine two such potential pathways—a plebiscitary election and an unauthorised referendum—through the lens of constituent power. We argue that both pathways are theoretically available if one accepts (as we argue) that the Scottish people is the bearer of constituent power. However, we conclude that there are significant obstacles dotting both potential pathways, and as such the only feasible route to internationally recognised statehood for Scotland is via political negotiation.

## 1. Introduction

A familiar spectre is haunting the UK—the spectre of Scottish independence. In June 2022, the then First Minister Nicola Sturgeon announced that a second independence referendum (Indyref 2) was to take place on 19 October 2023.^1^ As was the case in the run-up to the first independence referendum in 2014 (Indyref 1), much of the debate was initially consumed by the question of whether the Scottish Parliament is competent to legislate on an independence referendum without an Order from Westminster under section 30 of the Scotland Act 1998 (section 30 Order) allowing it to do so.^2^ In October 2022, the UK Supreme Court unanimously held that the Scottish Parliament does not have the power to legislate for Indyref 2 without a section 30 Order.^3^ The UK government is currently unwilling to make the relevant Order, with the result that the Scottish and UK governments are at an impasse. Sturgeon’s successor, Humza Yousaf, has set out the proposed timetable for his party to finalise its ‘independence strategy’.^4^ It is the optimum time, therefore, to turn our minds to the next question that poses itself: absent a section 30 Order, are there any other pathways to Scottish independence?

Scholarly opinion has tended to deny the existence of such pathways. For instance, according to Andrew Tickell, there is ‘no magic bullet’^5^ that would allow Scots to vote on independence without the UK government’s approval. Similarly, Christopher McCorkindale and Aileen McHarg contend that there ‘are no legal short cuts’^6^ to Scottish independence outside of a section 30 Order or legislation by the UK Parliament. However, one potential pathway for achieving Scottish independence has not yet been fully explored in the literature: invocation of the Scottish people’s constituent power, that is, the power to create its own constitutional order. In this article, we analyse this idea and conclude that it is unsuitable for bringing about effective statehood for Scotland, thus adding more weight to the finding that there is indeed ‘no magic bullet’ for Scottish independence outside an agreement between the two governments.

It is worth noting that the concept of constituent power is not without its critics, some of whom are concerned about what they see as its arbitrary character and its potential for despotic abuse.^7^ However, that has not prevented others from invoking the concept in the context of Scottish independence. It has not only emerged in scholarly debates, but also underpins the political proposals of some Scottish nationalists.^8^ And with the debate on Scottish independence still ongoing, we can expect the references to constituent power to become ever more frequent. For these reasons, we propose to take the concept seriously and assess which avenues for pursuing independence it opens up and what their drawbacks are.

There are many different theories of constituent power, but we will focus here on what we take to be the dominant approach, which views constituent power as a revolutionary power that allows its bearer to create a new constitution, even if it has to go against an existing constitution to do so.^9^ We focus on this theory not because we find it normatively attractive, but because its dominance will likely make it the most frequently invoked theory in the run-up to a potential Indyref 2. For the sake of clarity, we should also emphasise that this article does not discuss whether the UK constitution can (or should) be reinterpreted or revised in a way that allows a path towards Scottish independence. Rather, by focusing on constituent power, we explore alternative pathways that go beyond, or are in conflict with, the UK constitution.

In fact, on the dominant approach, exercises of constituent power are often characterised as ‘extra-legal’ or even ‘political’.^10^ This is because they operate outside a given constitution and are therefore seen as operating outside of law. As such, it could be said that the Scottish people’s constituent power and the alternative pathways to independence it may open up can only be assessed in virtue of their moral or political legitimacy, but not their lawfulness. However, while assessments of legitimacy play an important role, they should not supplant a juridical analysis,^11^ for the question of lawfulness is still at play in moments when constituent power is exercised, even if what is constitutional and lawful is up for grabs. After all, the outcome of a successful exercise of constituent power is a new constitution and a new ‘legality’, which can serve to retrospectively legalise the procedures that enabled it. For this reason, we propose that even though an exercise of constituent power by the Scottish people would violate the UK constitution, it could nevertheless be viewed as what we call a ‘quasi-legal’ pathway to independence.

By ‘quasi-legal’, we understand a pathway that would be illegal^12^ under the current UK constitution, but retrospectively validated under a new Scottish constitution. The idea of retrospective legal validation of constitution-making processes that are considered illegal under an existing constitution is not new, of course. As Hannah Arendt noted, it describes what some of the earliest thinkers of constituent power tried to make sense of: that those getting together to form a new constitution tend themselves to be ‘unconstitutional’.^13^ This unconstitutionality is overcome—in the eyes of those endorsing the new constitution, but not those defending the old constitution—with the ‘laying down’ of the new ‘fundamental law’,^14^ for only once the new constitution is in place can it give its blessing to its own creation. To use an analogy, the situation that would arise with the creation of a new Scottish constitution is comparable to Schrödinger’s cat: an exercise of constituent power by the Scottish people can be both unconstitutional (from the point of view of the UK constitution) and constitutional (from the point of view of a future Scottish constitution that retrospectively legalises its creation).^15^ What is legal about the quasi-legality of exercises of constituent power, then, is their legality from the point of view of a potential future Scottish constitution.

This possibility of a quasi-legal path to Scottish independence has at least two important implications. First, it reveals that a potential exercise of constituent power by the Scottish people cannot be fully understood in terms of moral or political legitimacy, but must also be considered in its legal dimension.^16^ Second, it shows that in theory—although, as we will argue, not in practice—scholars such as McCorkindale and McHarg could be wrong that there are ‘no legal short cuts’ to Scottish independence outside a section 30 Order.

To examine these implications, we focus on two concrete alternatives to a section 30 Order that have been mooted and that could be interpreted as exercises of constituent power: a plebiscitary election and an unauthorised referendum.^17^ The first alternative—a plebiscitary election—was Sturgeon’s Plan B.^18^ The second alternative—a referendum held without permission from the UK government—was consistently rejected by Sturgeon.^19^ However, others, including members of the SNP,^20^ have contemplated adopting this approach.^21^ It remains to be seen in which direction Yousaf will lead his party and thus both alternative pathways warrant consideration.

As we will show in this article, both a plebiscitary election and an unauthorised referendum can, in principle, be quasi-legal pathways to Scottish independence from the point of view of constituent power. However, as we will argue, they are inadvisable alternatives. This is because they are dotted by political obstacles that mean that internationally recognised statehood is unlikely to await at the end of either of these paths.

Our argument unfolds in four sections. Section 2 provides an overview of the concept of constituent power and argues that the Scottish people can be seen as its bearer. In section 3, we look at the first potential pathway, asking if and on which conditions the Scottish people can express its constituent power through a plebiscitary election. In section 4, we turn to the second pathway and discuss whether an unauthorised referendum can express the Scottish people’s constituent power. Section 5 reveals the political constraints that counsel against adopting either of these two alternative pathways. The concluding section recapitulates the article’s main insights and proposes a more practical way forward by way of political negotiation.

## 2. The Scottish People’s Constituent Power: A Primer

To assess the implications of constituent power in the context of Indyref 2, it is necessary to first outline what constituent power is, especially because it is not as popular a concept in British constitutional debates as it is elsewhere.^22^ Building on this outline of constituent power, we then establish that the Scottish people can be seen as its bearer.

Starting with the first point, according to the dominant approach on which we focus here, constituent power is defined as the power to create a constitution. It is a power that belongs to a people who generally relies on representatives such as constituent assemblies to exercise it on its behalf. Many trace the dominant approach to the French 18th-century theorist Emmanuel Joseph Sieyès, who articulated the distinction most clearly between the people’s constituent power (*pouvoir constituant*) and the constituted powers (*pouvoirs constitués*).^23^ The latter are the powers created by a constitution and include the common branches of government—the executive, legislative, and judicial branches—as well as other public institutions. Importantly, by contrast to the constituent power, constituted powers derive their authority from a constitution and thus cannot validly claim powers not conferred by it. Sieyès developed this distinction in the context of the French Revolution, and constituent power is still today primarily understood as a revolutionary power that is exercised in moments when a people gives itself a new constitution.^24^ In such ‘constitutional moments’, to use Bruce Ackerman’s words, the people stops pursuing ‘normal politics’ within the existing constitutional order and instead shifts to a different mode—‘higher lawmaking’^25^—which involves reshaping the constitutional order in fundamental ways.

McHarg’s work helps us understand how the theory of constituent power applies to Indyref 2. Writing in the context of Indyref 1, McHarg argued that that referendum constituted a ‘constitutional moment’.^26^ That is, it was a moment when normal politics gave way to higher lawmaking. As she notes, this is because the referendum could have (but did not in the event due to its result) ‘usher[ed] in a new constitutional era for Scotland (and, by implication, the rest of the UK)’.^27^ Her assessment strikes us as correct, and it suggests that Indyref 2 would represent a constitutional moment, too. This is despite the fact that declaring independence and creating a new constitution are not the same thing. Strictly speaking, only the latter is an exercise of constituent power. However, in contexts where a people first needs to break free from a state to be able to create its own constitution, the two cannot be easily separated. The constitutional moment can span both events, becoming effectively a constitutional period.^28^ Indeed, in debates on Indyref 1 and 2, independence has already generally been treated as a first and necessary step before the creation of a new constitution for Scotland.^29^

Independence referenda are a paradigmatic way of exercising constituent power.^30^ However, referenda and constituent power should not be equated, for two reasons. First, there can be constituent power without referenda. Sieyès himself did not consider referenda necessary for a nation to exercise constituent power. In fact, when France discussed the creation of a new constitution in 1789, he was opposed to the idea of having the constitution ratified by popular vote. Instead, he insisted that constituent power had to be exercised by the people’s representatives in the National Assembly.^31^ Another important proponent of the dominant approach, Carl Schmitt, also rejected the idea that constituent power must be exercised through referenda. For example, he held that constituent power could be expressed through ‘public opinion’^32^ or through constituent assemblies that are unaccompanied by referenda.^33^ That constituent power can be exercised without referenda is also borne out by practical examples of constitutions that came into existence without referenda. For instance, the Indian Constitution has been described as a product of constituent power, even though it did not involve people voting in a referendum.^34^

Second, there can be referenda without constituent power. To see this, recall Sieyès’s distinction between constituent and constituted powers. The latter include *all* powers created by the constitution, including powers given to the people. In many states, these include the people’s power to vote on constitutional reforms in referenda. Joel Colón-Ríos has proposed the helpful distinction between *constitutional* and *constituent* referenda to make sense of this fact.^35^ Constitutional referenda are referenda in which a people vote on a constitutional amendment as part of a constitutionally established procedure. Here, the people only exercises constituted power. By contrast, the people can only exercise constituent power in constituent referenda. Colón-Ríos draws on Schmitt to explain that constituent referenda are characterised by their altering ‘the material constitution’, that is, ‘the constitution’s fundamental content’.^36^ Hence, when a people votes in referenda that do not change the constitution’s fundamental content and are therefore not constituent referenda, it does not exercise constituent power.^37^ However, not even every constituent referendum necessarily expresses constituent power. A constituent referendum does not express constituent power when its results do not truly express the will of the people as the bearer of constituent power, as happened, for example, in the sham constituent referenda held in September 2022 in the Ukrainian regions of Luhansk, Zaporizhzhia, Kherson, and Donetsk.^38^

Disentangling referenda and constituent power reveals that holding an independence referendum sanctioned by a section 30 Order would not guarantee the exercise of constituent power. Even more relevantly for our purposes, it shows that, in principle, the Scottish people could exercise constituent power without having to act through a referendum.^39^ As the bearer of constituent power, the Scottish people would be free to exercise that power in other ways, including by delegating its exercise to bodies other than the Scottish Parliament or government. Hence, even if the Scottish Parliament were prohibited from passing the Independence Bill providing for Indyref 2 for want of a section 30 Order, the Scottish people could still exercise its constituent power and pursue independence by other means.

Whether the Scottish people can exercise constituent power of course necessitates it being the bearer of such power. The Scottish people is different from the French people in the 18th century in that Scotland is part of a larger state: the UK. Could it therefore not be objected that the idea of applying constituent power to the Scots is a non-starter because they hold, at best, a share of the constituent power that belongs to the UK people as a whole? This objection is based on two incorrect assumptions: first, that constituent power is held only at state level; and second, that the UK is a unitary state (ie a state with a single source of constitutional authority based in the Westminster Parliament).^40^

The first assumption—that constituent power is held only at the level of the state—has come under attack in recent decades. This assumption is challenged by those who claim that *supra-*state entities such as the European Union can manifest constituent power.^41^ In addition, it is under pressure from those who argue that *sub*-state entities such as federated states and devolved territories also have a claim to constituent power. Stephen Tierney has been one of the most prominent authors to make the latter point.^42^ Tierney takes issue with the widespread focus on unified peoples and territories as this ignores ‘sub-state national societies within plurinational states’.^43^ According to him, plurinational states are made up ‘of a plurality of territorially concentrated, potentially self-governing societies, which are possessed of a desire for specific constitutional recognition as such’.^44^ In contrast to uninational states, plurinational states are founded as ‘a union of pre-existing peoples subsequent to which sub-state national societies within the state continued to develop as discrete *demoi*’.^45^ As he notes, the existence of sub-state nations does not exclude the simultaneous existence of an overarching state *demos*. However, it does mean that there are nations present within the state that constitute

polities which are in fact comparable to the state in the way they offer, or have the potential to offer, an effective site for many, if not all, of those functional and indentificatory [*sic*] roles which the state plays in the life of the citizen.^46^

As Tierney points out, many plurinational constitutions do not give sub-state nations a constitutional right to pursue independence, but that does not mean that these nations cannot be bearers of constituent power.^47^

The second assumption—that the UK is a unitary state—is no less problematic.^48^ This is for two related reasons: the origins of the UK as a union state and the importance of devolution today. First, the historical relationship between Scotland and England is different from that between Wales and England or Ireland and England, among other things because England never conquered Scotland.^49^ Instead, the two nations formed a voluntary union with the Treaty of Union 1707.^50^ The preservation of Scotland’s independent legal system is just one example of Scotland’s unique position in the union. Second, the assumption also proves problematic considering the advanced state of devolution today.^51^ As McHarg points out, the idea that the Scottish Parliament is not only the expression of an Act of the UK Parliament but of ‘an understanding of the UK as a union state in which Scots enjoyed a right to self-government’^52^ is reinforced by the fact that the Scots approved its creation by referendum in 1997 with a large majority of 74.3%. Since then, Scottish devolution has developed to such an extent that Scotland is now ‘amongst the most autonomous substate regions in the world’.^53^ For these reasons, McHarg rightly argues that ‘the unitary version of the territorial constitution does not do justice to the significance of devolution, particularly in Scotland’.^54^ Interestingly, the UK government seems to have effectively endorsed this view when recognising on several occasions that the Scots have a right to self-determination, including in the Edinburgh Agreement (the agreement between the UK and Scottish governments to issue a section 30 Order to enable Indyref 1).^55^ Even prior to that, several prime ministers explicitly acknowledged the Scottish people’s right to self-determination.^56^

Self-determination is not, however, the same as constituent power. Self-determination is the broader concept and encompasses both the right of a people to have autonomy in political, economic, social, and cultural matters within a state (internal self-determination)^57^ as well as, in certain cases, a right to secession from a parent state (external self-determination).^58^ Constituent power is the narrower concept and describes the power to create a constitution. That power can overlap with external self-determination (when, as noted above, secession is a first and necessary step before a constitution can be created), but it can also occur outside secessions in cases when states witness the creation of a new constitution for their entire territory (as happened in France with the Revolution). The UK government’s recognition of Scottish self-determination relates to internal self-determination rather than external self-determination or constituent power. However, even that more limited recognition is sufficient to support the view that the UK is a union state consisting of different peoples. The conclusion that the UK should not be viewed as a unitary state follows regardless of whether one thinks that it was a union state since 1707 or that it has become one more recently because of devolution.^59^ Indeed, one might even agree with both views and argue that the UK was a union state from the start that has in recent decades seen a reawakening of Scotland’s dormant—but ever-present—claim to constituent power.^60^

For these reasons, we should reject the two assumptions on which the objection that the Scottish people cannot be seen as a bearer of constituent power rests: constituent power can be held at sub-state level, and the UK has such a sub-state level, with Scotland benefiting from recognition of its nationhood both for historical and devolution-related reasons. All these are strong considerations suggesting that the Scottish people should indeed be viewed as the bearer of constituent power, as many scholars have concluded.^61^

What follows from this? According to Tierney, that a sub-state nation like Scotland has a claim to constituent power does not necessarily mean that secession is made a priority.^62^ Instead, sub-state nationalists often first try to work within existing constitutional channels to protect their nation’s interests.^63^ Only when these formal channels are blocked do nationalists resort to challenging the constitutional order as such by claiming constituent power for themselves. As we noted above, such challenges are unconstitutional from the point of view of the existing constitution (assuming, as we do here, that the formal channels are blocked). But that qualification misses the full picture. As we saw, the dominant approach to constituent power views exercises of that power as ‘extra-constitutional’ in that they operate outside—or even against—the old constitution to create a new constitution. If it can successfully be established, such a new constitution creates its own new legality, which can then retrospectively legalise the means used to bring it into existence.^64^ As noted above, we use the term ‘quasi-legal’ to describe this phenomenon.

This framework explains two important things. First, it explains why invocations of the orthodox doctrine of parliamentary sovereignty to rebut Scottish constituent power cannot settle the matter. That doctrine—and the related idea that the word of the Scotland Act is final—relies on an understanding of constitutionality that is opposed to that of proponents of Scottish constituent power, according to which breaks with existing constitutional rules and principles can be(come) legal. Second, it explains why the UK Supreme Court, since it was invited to focus on the constitution’s formal channels, did not address the question of Scottish constituent power, which is based on a different way of viewing constitutionality.^65^ Indeed, in our view, constituent power would have been a foolhardy concept for counsel to rely on because quasi-legality would not fulfil the crucial goal for those who desire independence—that is, to achieve internationally recognised statehood.

## 3. Can a Plebiscitary Election Express the Scottish People’s Constituent Power?

Having established that the Scottish people can be seen as a bearer of constituent power and that constituent power can in principle be exercised by other means than referenda, we are now able to address the question of whether there are any other pathways to independence available to the Scottish people that, if not legal, are at least quasi-legal. In this section, we focus on whether, and on what conditions, a plebiscitary election would constitute such a pathway.

As noted in the Introduction, holding a plebiscitary election as a ‘de facto referendum’ was Sturgeon’s Plan B.^66^ However, should Yousaf wish to pursue this plan, many questions still need to be resolved. The Scottish Sovereignty Research Group (SSRG), a pro-independence think tank, explored this option as one of six ‘routes to independence’. According to the SSRG, a plebiscitary election can be used to ‘seek a democratic mandate to declare independence or to negotiate independence’.^67^ One way of doing this would be by treating the plebiscitary election as an expression of constituent power. To ensure that the relevant mandate is as clear as possible, the SSRG advises pro-independence parties to focus on a sole item in their manifesto: ‘that should they succeed in winning a majority of Scottish seats then that would provide them with a mandate to declare independence’. As such, it would be ‘wise for these parties to exclude any other policies in their manifestoes in order to avoid any confusion about the outcome should they win’.^68^

Can the Scottish people exercise its constituent power through a plebiscitary election and if so, how? The little scholarship that exists on this question appears largely sceptical.^69^ Pravar Petkar, who has considered the question from a constituent power perspective, finds that a plebiscitary election would not amount to an exercise of constituent power:

Such a vote, entailing the election of representatives rather than a direct vote on a policy matter, is less participatory than a referendum and so is better viewed as an election for the Scottish *constituted* powers rather than an expression of constituent power. This will be especially so if other Scottish parties make manifesto commitments on other policy issues, diluting the extent to which the election concerns a matter of constitutional change.^70^

McCorkindale and McHarg, while not focusing on constituent power, have also taken issue with the proposal, arguing that a mandate gained through an election can only have political, not legal relevance. Furthermore, they point out that any such mandate would be ‘highly ambiguous’:

how clear does a manifesto promise have to be; is a majority of seats or of votes required (and can these be aggregated from more than one party); and which elections are relevant—to the UK Parliament, which holds the legal competence to dissolve the Union, or the Scottish Parliament, from which the Scottish Government’s authority derives?^71^

These authors make valid points. However, they do not show that the Scottish people cannot express constituent power through a plebiscitary election. To see this, we need to distinguish two separate issues: *whether* a plebiscitary election can express constituent power and *on what conditions* it can do so. Answering these questions helps us establish whether exercising constituent power through a plebiscitary referendum is possible.

We start with the first question: can a people ever express its constituent power through plebiscitary elections? If it could, then this would be of quasi-legal and not just political relevance. This is because constituent power is the power to create a constitution, which, as noted above, is of not just political but also juridical significance. By arguing that a plebiscitary election could only be politically relevant, McCorkindale and McHarg imply that constituent power cannot be exercised through such an election. Perhaps this is because, in her work discussing constituent power, McHarg has only considered constituent power in the context of referenda.^72^ However, as we explained, constituent power and referenda should not be equated: constituent power can be, and has been, expressed by other means than referenda.

This leaves the question of whether plebiscitary elections are such an ‘other means’ by which constituent power can be expressed. There is no reason in principle to think that they are not. Leading adherents of the dominant approach to constituent power would not have ruled out plebiscitary elections. Sieyès, for example, was adamant that, to express its constituent power, the people cannot be bound by any particular forms. In the context of the French Revolution in 1789, he proposed that the people should commission extraordinary representatives with the exercise of constituent power.^73^ However, he was also open to other forms, arguing that:

It would be ridiculous to suppose that the nation [ie the people] itself was bound by the formalities or the constitution to which it had subjected those it had mandated. If a nation had to wait for a positive mode of being in order to become a nation, it would simply never have had an existence.^74^

The same is true for Schmitt, who contemplated numerous ways in which a people could exercise constituent power, including through acclamation (‘the assembled multitude’s declaration of their consent or disapproval’),^75^ public opinion, and plebiscites. As Yaniv Roznai summarises the dominant approach, constituent power is unlimited ‘at least in the sense that it is not bound by previous constitutional rules and procedures’.^76^

If, according to the dominant approach, a people is not bound by any particular forms, then it must follow that it can express its constituent power through elections.^77^ This follows *a fortiori* if, like Schmitt, one accepts less democratic forms of expression, such as the gauging of public opinion. If they are well run, elections provide a reasonable degree of democratic legitimacy. That is not to say, of course, that every plebiscitary election can or should be viewed as an exercise of constituent power. But it does mean that, in principle, a plebiscitary election could be a vehicle for constituent power. The real question is therefore not whether a plebiscitary election can express constituent power—it can. It is on which conditions it can do so. And to answer that question, we need to move from the issue of principle to that of practicality.

From a practical perspective, there are numerous hurdles that need to be overcome for a plebiscitary election to be an authentic exercise of constituent power. Some of these conditions may prove so difficult to meet that they make this an unattractive path. As McCorkindale and McHarg note, several ambiguities need to be resolved, including which elections are relevant, what majority is required and how clear a manifesto must be. Resolving all these ambiguities is not something we can do in this article. However, we can identify two main yardsticks that will be relevant for anyone attempting to clarify these matters. If an election is to express the Scottish people’s constituent power, then it must leave as little doubt as possible, first, on *what* the Scottish people’s will is and, second, that this *is* indeed the people’s will (rather than just a small subsection thereof). No election may achieve absolute clarity on either of these points, so one will necessarily have to determine what the relevant thresholds are that, if met, allow identifying the ‘what’ and ‘is’ with sufficient clarity.^78^

Take, for example, the question of whether a UK general election would be more suitable than a Scottish Parliament election. An answer to this will depend on which of these elections can more clearly express *what* the Scottish people wants and that it *is* something it wants. Given that, from a constituent power perspective, the focus will need to be on what the Scottish people wants (rather than what peoples in England, Wales or Northern Ireland want), there is no obvious reason why a UK general election would be more suitable than a Scottish Parliament election.^79^ A UK general election would only be more suitable if one assumed that we are dealing with a unitary state, as this would make it necessary for the UK people as a whole to be involved. However, as we saw above, that assumption is problematic. Equally, however, there is no obvious reason why a UK general election would be any less suitable. As long as one only focuses on whom the Scottish people elects, one could also determine its will with sufficient clarity in a UK general election. What this shows is that, if we use the two yardsticks mentioned above, then the difference between the two types of elections seems insignificant when it comes to the question of identifying the ‘what’ and ‘is’. Indeed, in 2020, the SNP suggested that the Scottish Parliament elections would be the venue for a *de facto* referendum,^80^ whereas in June 2022, Sturgeon envisaged the next UK general election as the place for such a referendum. It appears Sturgeon only pivoted to the next UK general election because it was due to happen sooner (no later than January 2025) than the next Scottish Parliament election (scheduled for May 2026).^81^ She may have then realised the potential pitfalls of choosing Westminster, such as the fact that 16- to 17-year-olds, EU nationals, and legally resident foreign nationals (all of whom may be more in favour of independence)^82^ would not be able to vote.^83^

What about the type of majority that is needed to express that this *is* an exercise of constituent power? The basic principle here is that the more votes go to a party committed to independence (on which we say more below), the clearer it will be that there *is* an exercise of constituent power. However, we also have to determine what the relevant threshold is that establishes that that *is* the case with sufficient clarity. In particular, would a simple majority be sufficient or is a supermajority necessary? In the case of referenda, which have served as expressions of constituent power elsewhere, simple majorities have generally been viewed as sufficient.^84^ However, given the fact that plebiscitary elections—unlike referenda—are not designed to express a people’s will on a policy matter, one could also argue that a supermajority is necessary to make up for the shortcomings of plebiscitary elections. At this stage, the question may therefore be posed as to how appropriate it is to harness the election format for a completely different purpose. While we do not have to decide this issue here, what seems clear is that although a simple majority may suffice, a supermajority would be less assailable because it would allow for a clearer expression of the people’s will when exercising constituent power in a form which is inherently less clear on policy matters than a referendum. Finally, should it be a majority (or supermajority) of votes or of seats?^85^ Using a UK general election rather than an election to the Scottish Parliament would avoid the potential added complication of how this question ought to be answered under the Additional Member System.^86^ If we again take referenda as our point of comparison, then it is arguable that a majority of votes, and not just seats, may be necessary.^87^

Another important question is how a party like the SNP could participate in an election in a way that turns that election into a *plebiscitary* election, that is, an election that clearly expresses what the Scottish people’s will is on a policy question (eg independence or no independence). The SNP and the SSRG are right in suggesting that having only one manifesto commitment (on bringing about independence) is necessary for clarity. But is it also sufficient? As Pravar warns, dilution could occur ‘if other Scottish parties make manifesto commitments on other policy issues’.^88^ It is true that dilution could occur if there are parties that run on a commitment of independence *as well as* other issues. It would be difficult to identify votes for such parties as clear votes for independence (and aggregate them with the SNP’s votes) given the possibility that citizens may have voted for them based on their commitments on other issues. However, it is unclear how any significant dilution could occur if other parties ran exclusively on manifesto commitments *other than* independence. In this case, if such parties only received the support of a minority of the Scottish people and the SNP received a sufficient majority on an independence-only commitment, it would be possible to determine with sufficient clarity what the Scottish people’s will on independence is.^89^ In fact, this would also hold if other parties ran on manifesto commitments *relating to* independence (whether for or against it): as long as the SNP received a sufficient majority on an independence-only commitment, it would not matter what other parties did and how successful they were. At present, the situation would be complicated by the fact that the SNP is in a power-sharing agreement with the pro-independence Scottish Green Party (SGP), which may be unwilling to omit environmental concerns from its manifesto.

Assume for the sake of argument that the SNP (or the SNP and the SGP) fight an election on the sole commitment to bring about independence and that they receive a sufficient majority of votes. Would that be enough to say that constituent power has been exercised in Scotland? Not necessarily, as other factors would be important, too. For example, the mere fact that a party has a certain commitment in its manifesto does not mean that every voter will be familiar with it.^90^ Some may simply vote for the SNP out of habit, or to reward them for one of their past manifesto commitments that had nothing to do with independence. Also, left-leaning Scottish voters may vote tactically for the SNP rather than, for example, Labour in an SNP/Conservative marginal seat.^91^ To get around this problem, there would have to be sufficient public debate in the run-up to the election—and coverage in a broad set of media—on the fact that the election is not a normal election but a plebiscitary election, at least as far as votes to the SNP are concerned.

Even if this was the case, however, establishing that the people’s decision is for independence could still be problematic if voter turnout is low,^92^ a problem which may well be more acute than usual. How does the tactical SNP voter in the marginal constituency vote if she is not in favour of independence? Does she bother voting at all? To have more clarity on this, compulsory voting could be introduced. However, as is true for meeting some of the other practical conditions mentioned, this would likely prove challenging considering that there is currently no majority support for compulsory voting,^93^ and that it could be perceived as too much of a change to the existing political system. Finally, it is worth emphasising that however clear a plebiscitary election is, it will likely never match a referendum, given that elections are not designed for policy questions.^94^

The above-mentioned practical obstacles would need to be overcome for a plebiscitary election to express the Scottish people’s constituent power. Those obstacles make this a thorny path to independence. In fact, some of the practical conditions may be so difficult to meet that constituent power may not materialise this way. However, this ultimately remains a matter of practical contingency, not theoretical possibility. As we have argued, a plebiscitary election could still in principle serve as a quasi-legal pathway to independence.

## 4. Can an Unauthorised Referendum Express the Scottish People’s Constituent Power?

Let us turn to the second alternative pathway to independence: an unauthorised referendum—sometimes also called a unilateral or ‘wildcat’ referendum. A referendum is ‘unauthorised’ when it lacks authorisation from the body on whose permission it ostensibly depends, as occurred in Quebec in 1995 and Catalonia in 2017. In the context of Scottish independence, the term ‘unauthorised referendum’ refers to a referendum conducted without authorisation from the UK government in the form of a section 30 Order or an Act of the UK Parliament. As mentioned, the SNP has not thus far proposed to conduct an unauthorised referendum. Sturgeon emphasised her ‘Government’s respect for the principles of rule of law and democracy’, explaining that ‘Respect for the rule of law means that a referendum must be lawful’.^95^ But what if the SNP, under its new leadership, wishes to explore the possibility that an unauthorised referendum is in fact a quasi-legal pathway to independence—authorised retrospectively, if you will, by a potential future Scottish constitution?

To see why this possibility should not be discarded, recall what we have said in the previous section about the forms through which constituent power can be exercised. As we saw, according to the dominant approach, the exercise of constituent power is not restricted to any particular procedures. Sieyès believed that this is a key difference between constituent power and constituted powers. Constituent power can be exercised how a people wants. By contrast, a constituted power is ‘nothing without its constitutive forms; it acts, proceeds, or commands only by way of those forms’.^96^ The UK Supreme Court seemed to have this latter paradigm in mind when it held that ‘statutory authority is needed’^97^ to regulate the date, question, franchise, spending rules and other formal aspects of an independence referendum. However, this runs contrary to the idea of constituent power. After all, as we saw, it is one of the defining marks of constituent power that it allows a people to create a constitution unbounded by the legal constraints of the existing constitutional order. As Mattias Kumm puts it, the ‘function of the idea of constituent power is to justify transformative political actions as exercises of legitimate authority that cannot be justified with reference to existing legal rules’.^98^ Think, for example, of the French Revolution—the context in which Sieyès developed his theory. His point was that the Third Estate formed a self-sufficient nation possessing constituent power, which it could exercise despite the resistance from the First and Second Estates and the King. Indeed, if the Third Estate had required the authorisation of the rulers at the time, it would likely never have been able to create a new constitution and put an end to the *ancien régime*. The purpose of constituent power is to provide a way out of such political stalemates.^99^ That is why it can be exercised even if—and especially if—the powers that be withhold authorisation.^100^ Accounts that try to answer the question of the constitutionality of Indyref 2 by focusing exclusively on the Scotland Act fall short because they miss that point. For the same reason, while the Supreme Court was right to note that a statutory basis for an independence referendum is an obvious way of ‘confer[ring] legitimacy upon the result’,^101^ such a basis is not necessary for constituent power to be exercised. Hence, while an exercise of constituent power can be authorised,^102^ such authorisation is neither necessary nor sufficient for it to be an exercise of constituent power, even if it can bolster its legitimacy.

There are two reasons that speak in favour of viewing unauthorised referenda as a means of expressing constituent power, and therefore a potential quasi-legal pathway to independence. First, it is arguable that unauthorised referenda are not only *compatible* with constituent power, but can be *more obvious* expressions of it than authorised referenda. The reason for this is that, in constitutions that provide for referenda as a form of constituted power, there is always the possibility that, when voting in a referendum, a people only expresses constituted power, not constituent power. As we saw above, Colón-Ríos introduced the distinction between constitutional and constituent referenda to emphasise this ambiguity. This issue cannot arise regarding unauthorised referenda. Due to their being unauthorised, they can only exercise constituent power, not constituted power. As such, they can be more obvious expressions of constituent power. To be sure, this does not mean that they necessarily express that power. As we saw above when considering the recent sham referenda in Ukraine, not all constituent referenda express constituent power.^103^ However, due to its ‘wildcat’ nature, an unauthorised referendum cannot be an exercise of constituted power.

Second, if one accepts our argument in the previous section that the Scottish people can in principle exercise constituent power through a plebiscitary election, then one should *a fortiori* also accept that it can do so through an unauthorised referendum. This follows because, when it comes to constituent power, plebiscitary elections are the hard case and referenda the easy one. As we saw, for a plebiscitary election to express constituent power, the stars need to align. Numerous practical conditions need to be met so that an election can authentically express constituent power because elections are not normally designed to express the people’s will on a policy question. Referenda do not run into this problem. Whether they are authorised or not, they can more easily express a people’s will on a question such as independence. That is why, as we noted above, referenda are generally viewed as a paradigmatic way of exercising constituent power.

To sum up, authorisation from the UK government is not required for a Scottish independence referendum to express the Scottish people’s constituent power. A unilateral referendum can, in principle, provide a quasi-legal pathway to independence, albeit not one which we would recommend, as we will explain in the next section.

## 5. The Political Constraints of Plebiscitary Elections and Unilateral Referenda

The previous sections have demonstrated that the Scottish people, as a sub-state nation, can pursue independence by exercising constituent power through a plebiscitary election or a unilateral referendum, provided the relevant conditions are met. However, just because these pathways are quasi-legal does not mean that they are advisable. Quasi-legal possibility (meaning the retrospective validity that a new Scottish constitution might bestow upon its process of creation) and political feasibility are two very different things. In this section, we use Catalonia as a case study to highlight the political obstacles that stand in the way of pursuing independence by means of plebiscitary elections and unauthorised referenda. Based on this, we will recommend a more conciliatory path between the Scottish and UK governments.

Both a plebiscitary election and an unauthorised referendum would face domestic political hurdles, but our focus in this section will be on the challenges they would face in international politics,^104^ which are more consequential when it comes to secession. As McHarg has pointed out, for advocates of Scottish independence, it is essential that the independence process is ‘legally and constitutionally robust, not only so that it can withstand domestic legal challenge, but also because that will be a crucial determinant of Scotland’s international standing in the event that it does become independent’.^105^ Compared to other nations, Scotland also has a more specific reason for taking international politics seriously: a large majority of Scots want to rejoin the European Union,^106^ and indeed this desire underpins the SNP’s aim to pursue a path to independence that is internationally acceptable. According to the Party’s 2019 manifesto, to follow an

[a]greed process means that no-one will be able to question the legitimacy of the referendum both here in Scotland and in the wider international community. For EU member states in particular, it will be essential to demonstrate that a referendum has been held legally and constitutionally.^107^

The crucial question, then, is whether Scottish independence achieved through either of the two pathways outlined above would be perceived as legitimate by the international community in general and the EU in particular.

Under international law, Scotland does not have a right to secede from the UK. International law provides for the self-determination of peoples (including in article 1(2) of the UN Charter). However, a right to secession is only recognised in exceptional circumstances because secessions that lack the consent of the parent state (either as a constitutionally guaranteed right or an *ad hoc* permission) are in tension with the fundamental principle of territorial integrity.^108^ The primary exception to this principle is colonised peoples seceding from their colonial parent states.^109^ Although more disputed, there is now also increasing recognition that in cases of severe human rights violations, even a non-colonised people can have a so-called ‘remedial right’ to secede from its parent state.^110^ However, the threshold is high for something to count as a severe human rights violation.^111^ Finally, as the Canadian Supreme Court observed in *Reference re Secession of Quebec*, some have proposed that peoples have a last-resort right to secede from their parent state if they are prevented from meaningfully exercising their internal self-determination in political, economic, social and cultural matters within that state.^112^ While some scholars have advocated granting a right to secession in an even broader range of circumstances,^113^ the prevailing view today is that, absent a consensual break-up, a right to secede is only available in colonial contexts, cases of severe human rights violations and, perhaps, cases where internal self-determination is blocked. This seems to paint a grim picture for Scottish independence, as none of these conditions are met in the Scottish case. Indeed, the Supreme Court was swift to reject the SNP’s argument that Scotland had a right to secede based on self-determination in international law.^114^

However, even if the Scots lack a *positive* right to secede from the UK, that does not mean that international law *forbids* Scottish secession. This is supported by the International Court of Justice (ICJ)’s 2010 Advisory Opinion on Kosovo, in which the Court was asked to determine whether Kosovo’s unilateral declaration of independence from Serbia was ‘in accordance with international law’.^115^ The Court answered that question in the affirmative, holding that ‘international law contains no applicable prohibition of declarations of independence’.^116^ The Court made clear that even if a people lacks a positive entitlement to secede, that does not mean that its secession necessarily violates international law. As long as it does not achieve independence in a way that violates *jus cogens*,^117^ it cannot be accused of breaking international law. This is because international law does not apply to such a people in the first place. According to the Court, the principle of territorial integrity is only binding on states.^118^ Since a seceding people is not (yet) a state, it cannot violate that principle. As Weller explains, the ICJ thus carved out a space for cases of ‘unprivileged secession … where an entity cannot rely on a positive claim to self-determination in the sense of a right to unilateral secession. However, its secession is not unlawful either.’^119^ If Scotland were to pursue such an ‘unprivileged secession’ from the UK, it too could not be accused of violating international law because it is only a nation, not a state. In contrast to a people that has a right to secede, however, other states could not support Scottish secession militarily or in other significant ways, nor could they recognise Scotland before it meets the criteria of statehood.^120^ This is because such support and recognition would amount to an external intervention into the UK’s territorial integrity.^121^

In short, under international law, Scotland has a lawful path to secession from the UK. But this path could lead into an international no man’s land where its new, independent statehood would likely not be recognised.^122^ The UK Supreme Court was therefore incorrect to say that the notion of self-determination ‘is simply not in play’ in the Scottish case.^123^ They were correct, however, to say that there are ‘insuperable obstacles’ in the way of harnessing that notion as a route to independence.^124^

That such an ‘unprivileged’ path to secession would indeed be politically undesirable is illustrated by the case of Catalonia, where separatists tried to achieve independence both through a plebiscitary election as well as a unilateral referendum.^125^ The Catalan government pursued the first path in the Catalan elections on 27 September 2015 after its years of lobbying the Spanish government to permit Catalonia to hold a referendum bore no fruit. Cast as a ‘plebiscitary election’,^126^ two of the three main parties fielding candidates in the election agreed on a common pro-independence platform called Junts pel Sí (Together for Yes).^127^ Together with another separatist party, Junts pel Sí won a majority of seats, but missed a majority of votes. Despite this, it claimed that the plebiscite was successful and that it handed Junts pel Sí a mandate to pursue independence from Spain.^128^ However, far from enabling it to make good on its manifesto promise, the plebiscitary elections failed to have the desired political effect. As Elisenda Casanas Adam has pointed out, ‘neither the Spanish authorities nor the EU conferred any significance on the outcome going further than that given to ordinary Catalan election results nor did they agree to open up any type of negotiations directed to discussing Catalan independence’.^129^ The same is true for the wider international community. To this day, no state has recognised Catalonia’s plebiscitary elections.^130^

Catalonia’s unilateral referendum fared no better. The Catalan government decided to pursue an independence referendum in 2017 despite the Spanish Constitutional Court having ruled unconstitutional and void the law of the Catalan Parliament on which the referendum was based.^131^ Because the Catalan government decided to go ahead with the referendum anyway, the Spanish government deployed more than 10,000 Spanish police officers in an attempt to prevent the referendum from taking place. The operation led to the arrest of civil servants and politicians and the searches of ministries and businesses, as well as the blocking of websites, the banning of public events and the lifting of postal secrecy to intercept referendum materials.^132^ Despite all these efforts, the referendum still went ahead because of volunteer organisations that effectively carried it out clandestinely.^133^ The referendum was approved by an overwhelming majority of 90% (albeit with a voter turnout of only 43%), leading then-Catalan President Carles Puigdemont to state that he had a mandate to declare independence from Spain—something a majority of members of the Catalan Parliament later formalised by way of resolution as a Declaration of Independence.^134^ The central Spanish government, which did not recognise the referendum, responded by temporarily suspending Catalonia’s autonomy and by dissolving the Catalan Parliament and dismissing its government.^135^ Numerous members of the Catalan government either fled their country or were prosecuted for sedition. Despite condemnation from international human rights NGOs, the Spanish Supreme Court sentenced nine of them to prison sentences ranging from nine to 13 years, which in turn resulted in protests and riots, with nearly 600 reported injuries.^136^ As in the case of the plebiscitary election, the international community responded unfavourably. The EU’s reaction was swift and uncompromising: the day after the referendum, the European Commission released a statement in which it invoked the Spanish Constitution, under which ‘yesterday’s vote in Catalonia was not legal’. The Commission made clear that it considered the unauthorised referendum to be a purely internal matter which did not merit the EU’s involvement, let alone recognition.^137^ Other states were similarly dismissive, and none have recognised Catalonia’s independence from Spain.^138^

It is important not to ignore the differences between Scotland and Catalonia. For example, in contrast to the Spanish government’s resistance to Catalan self-determination, the UK government has long accepted that Scotland is a political nation with a right to internal self-determination. Also, unlike the Spanish Constitution, the UK constitution does not explicitly rule out Scottish secession.^139^ Furthermore, because of Brexit, Scotland would not face the problem that its former parent state could veto its accession to the EU in the European Council. Given these differences, we do not take a stance on whether Catalonia (or other polities, such as Quebec) had or has a valid claim to constituent power. For our purposes, it is sufficient that some believe it did.^140^

Despite these differences, Catalonia’s experience holds important lessons for Scotland. The first, and main, lesson is that an agreement between Scotland and the UK government will be indispensable for independence to be politically feasible. This holds, first of all, for Scotland’s recognition by the international community generally. As Weller has pointed out, without a ‘divorce by agreement’, Scotland would share Catalonia’s fate of non-recognition and thus ‘fall into a position of international legal limbo’.^141^ The same holds more specifically if Scotland wants to rejoin the EU. Here, too, an agreement with the UK would be vital. This is because without it, Scottish accession would likely be vetoed by some Member States, including Spain, Romania, Slovakia, Cyprus, and Greece. These states have refused to recognise Kosovo’s independence—despite its backing by the UN and its recognition by 117 other states—on the ground that Kosovo’s parent state (Serbia) has not consented to its independence.^142^ These problems could be avoided if Scotland seceded with the UK government’s permission, as this would effectively grant it a right to secede, which in turn would make it politically difficult for these Member States and the international community more generally to withhold recognition of Scottish statehood.^143^ What is more, an agreement with the UK would also be an essential condition for Scotland’s membership in the UN, which does not tend to grant membership in cases of unilateral secession.^144^

The second lesson for advocates of Scottish independence is that, instead of helping their cause, pursuing independence through a plebiscitary election or an unauthorised referendum may actually set their movement back. According to José Luis Martí, the unilateral Catalan approach was a strategic ‘disaster’ because it produced ‘a very strong opposition and negative opinion from the rest of Spain that is counterproductive for seeking a negotiated agreement’.^145^ Inside Catalonia, too, there appears to have been a setback for separatists. Opinion polls on support for independence show an 8% drop compared to 2017, to 41%, with only 11% in favour of another unilateral process. What is more, internal rifts among Catalan nationalists mean that the path forward is less clear than ever.^146^ Part of the reason for this setback within Catalonia may be the fact that Catalonia’s leaders promised Catalans something that the plebiscitary election and unilateral referendum could not deliver: politically legitimate independence that leads to recognition of statehood by the international community. As Casanas Adam has pointed out, the same would likely be true for Scotland.^147^

For these reasons, proponents of Scottish independence should not be fooled by the fact that international law does not forbid independence achieved through a plebiscitary election or an unauthorised referendum: independence achieved in these ways would be a Pyrrhic victory because it would impede Scotland’s recognition as an international state and potentially set back independence at home, too.

## 6. Conclusion

In the light of the Supreme Court’s judgment that legislation enabling Indyref 2 is a reserved matter for which the Scottish Parliament requires authorisation from the UK government, we analysed two potential alternative pathways to independence from the underexplored perspective of the Scottish people’s constituent power. After clarifying the relationship between constituent power and referenda and arguing that the Scottish people, as a sub-state nation, can be viewed as a bearer of constituent power, we showed that plebiscitary elections and unilateral referenda could in principle be quasi-legal pathways to independence, in the sense that a new Scottish constitution could bestow retrospective legality on the process of its creation. To date, contemporary scholarship has failed to take seriously the implications of the Scottish people’s constituent power. However, as we stressed, just because something is quasi-legal does not mean it is politically advisable. Using Catalonia’s negative experience with plebiscitary elections and unilateral referenda as a cautionary tale, we argued that in the Scottish context, too, pursuing either of these pathways would likely not lead to internationally recognised statehood.

For these reasons, Tickell is correct that there is ‘no magic bullet’^148^ that would allow Scots to achieve independence without the UK government’s consent. The reason for this is not that there is no way around the Scotland Act—there is. The reason is that if Scots want to achieve proper independence with recognition from the EU and the international community, then the only path forward is reaching a divorce agreement with the UK government by way of political negotiation.^149^ That such an agreement is not beyond the realms of possibility is evidenced by the fact that the Scottish and UK governments already managed to achieve such a ‘process of statesmanship’^150^ with the Edinburgh Agreement, which paved the way for Indyref 1.

Indyref 1 may have been intended to be a ‘once in a generation’ event,^151^ but the Scottish government has a compelling argument that there have been ‘material changes in circumstances’^152^ since then—the most notable being Brexit. In 2018, McHarg wondered what ‘degree of territorial variation’ the UK could endure, and predicted that ‘the break-up of the UK seems a more likely prospect than its significant reform’.^153^ In the intervening years, relations have worsened rather than improved. For example, the Scottish government’s opposition to the potential repeal of the Human Rights Act 1998 as well as its general greater commitment to rights protection (including economic, social, cultural, and environmental rights) provides more evidence for the divergence between Scotland and the rest of the UK (or at least England).^154^

No UK government wishes its place in the history books to be as the government that facilitated the break-up of the Union. But the current impasse may only serve to deepen the wounds in the Union.^155^ That said, it is far from certain that the outcome of Indyref 2 would be any different from Indyref 1.^156^ What is certain, however, is that only a ‘gold standard’^157^ Indyref 2 has the potential to put an end to the debate, one way or another.

## Footnotes

1 Scottish Parliament, ‘Official Report, Meeting of the Parliament (Hybrid), 28 June 2022’, 15.

2 See eg Elisenda Casanas Adam, ‘An Unanswered or Unanswerable Question? The Scottish Parliament’s Competence to Legislate for an Independence Referendum for Scotland’ (2017) 54 Revista Catalana de Dret Públic 81; Stephen Tierney, ‘The Lord Advocate’s Reference: Referendums and Constitutional Convention’ (*UK Constitutional Law Association Blog*, 4 October 2022) <https://ukconstitutionallaw.org/2022/10/04/stephen-tierney-the-lord-advocates-reference-referendums-and-constitutional-convention/> accessed 16 June 2023; Leah Trueblood, ‘What Is the “Purpose” of the Scottish Independence Referendum Bill?’ (*UK Constitutional Law Association Blog*, 11 October 2022) <https://ukconstitutionallaw.org/2022/10/11/leah-trueblood-what-is-the-purpose-of-the-scottish-independence-referendum-bill/> accessed 16 June 2023.

3
*Reference by the Lord Advocate of Devolution Issues under paragraph 34 of Schedule 6 to the Scotland Act 1998* [2022] UKSC 31.

4 SNP, ‘SNP Convention on Independence’ <www.snp.org/events/snp-convention-on-independence/> accessed 20 June 2023.

5 Andrew Tickell, ‘No Magic Bullets: Legal Issues in Achieving Independence and a Referendum’ in Gerry Hassan and Simon Barrow (eds), *A Better Nation: The Challenges of Scottish Independence* (Luath 2022) 85.

6 Christopher McCorkindale and Aileen McHarg, ‘Constitutional Pathways to a Second Scottish Independence Referendum’ (*UK Constitutional Law Association Blog*, 13 January 2020) <https://ukconstitutionallaw.org/2020/01/13/chris-mccorkindale-and-aileen-mcharg-constitutional-pathways-to-a-second-scottish-independence-referendum/> accessed 16 June 2023.

7 See eg Hannah Arendt, *On Revolution* (Penguin Books 1965) 163; David Dyzenhaus, ‘Constitutionalism in an Old Key: Legality and Constituent Power’ (2012) 1 GlobCon 229; Lars Vinx, ‘The Incoherence of Strong Popular Sovereignty’ (2013) 11 ICON 101; Lars Vinx, ‘Ernst-Wolfgang Böckenförde and the Politics of Constituent Power’ (2019) 10 Jurisprudence 15. For an overview of different critiques, see Sergio Verdugo, ‘Is It Time to Abandon the Theory of Constituent Power?’ (9 March 2023) <https://papers.ssrn.com/sol3/papers.cfm?abstract_id=4162160> accessed 16 June 2023.

8 See eg Stephen Tierney, ‘“We the Peoples”: Constituent Power and Constitutionalism in Plurinational States’ in Martin Loughlin and Neil Walker (eds), *The Paradox of Constitutionalism* (OUP 2007); Aileen McHarg, ‘The Independence Referendum, the Contested Constitution, and the Authorship of Constitutional Change’ in Klaus Peter Müller (ed), *Scotland 2014 and Beyond—Coming of Age and Loss of Innocence?* (Peter Lang 2015); Pravar Petkar, ‘Consultative Referendums and Constituent Power in the UK’ (*UK Constitutional Law Association Blog*, 5 July 2022) <https://ukconstitutionallaw.org/2022/07/05/pravar-petkar-consultative-referendums-and-constituent-power-in-the-uk/>. On political proposals, some of which are (implicitly) based on constituent power, see Scottish Sovereignty Research Group, ‘Routes to Independence’ <https://web.archive.org/web/20230327234500/https://scottishsovereigntyresearchgroup.org/routes-to-independence/> accessed 16 June 2023.

9 For a representative account, see Andreas Kalyvas, ‘Constituent Power’ in JM Bernstein, Adi M Ophir, and Ann Laura Stoler (eds), *Political Concepts: A Critical Lexicon* (Fordham UP 2018). See also Carl Schmitt, *Constitutional Theory* (Jeffrey Seitzer tr, Duke UP 2008); Antonio Negri, *Insurgencies: Constituent Power and the Modern State* (Maurizia Boscagli tr, University of Minnesota Press 1999). Andrew Arato calls the dominant paradigm ‘sovereign constituent power’: Andrew Arato, *The Adventures of the Constituent Power: Beyond Revolutions?* (CUP 2017) 45. For overviews of different interpretations of constituent power, see Lucia Rubinelli, *Constituent Power: A History* (CUP 2020); Joel Colón-Ríos, *Constituent Power and the Law* (OUP 2020).

10 For an overview, see Colón-Ríos, *Constituent Power and the Law* (n 9) 1–28.

11 See also Andreas Kalyvas, ‘Popular Sovereignty, Democracy, and the Constituent Power’ (2005) 12 Constellations 223, 232 (arguing that constituent power is a ‘juridical category *par excellence*’).

12 For the purposes of this article, we use the terms ‘illegal’ and ‘legal’ synonymously with ‘unconstitutional’ and ‘constitutional’, respectively.

13 As we discuss in s 4, constitutions can authorise the creation of a new constitution. But these are not the cases Arendt has in mind here: Arendt (n 7) 175–6. The idea of retrospectivity is also prominent in constituent power scholarship adopting a relational approach, see Nico Krisch, ‘*Pouvoir Constituant* and *Pouvoir Irritant* in the Postnational Order’ (2016) 14 ICON 657, 660; Hans Lindahl, ‘Possibility, Actuality, Rupture: Constituent Power and the Ontology of Change’ (2015) 22 Constellations 163. Note that Lindahl distinguishes between retrospectivity and retroactivity: Hans Lindahl, ‘A-legality, Representation, Constituent Power: Reply to Critics’ (2019) 21 Ethics & Politics 417, 471. See also Jason Frank, *Constituent Moments: Enacting the People in Postrevolutionary America* (Duke UP 2010) 10. Because we focus on the dominant (non-relational) approach, our use of the term ‘retrospective’ differs from the usage in this scholarship insofar as the dominant approach is not concerned with the retrospective (or retroactive) identification of the constituent subject.

14 Arendt (n 7) 176.

15 For a different use of Schrödinger’s cat which inspired ours, see Pravar Petkar, ‘A Second Scottish Independence Referendum and Schrodinger’s Sub-State Constituent Power’ (*IACL-IADC Blog*, 29 November 2022) <https://blog-iacl-aidc.org/new-blog-3/2022/11/29/a-second-scottish-independence-referendum-and-schrodingers-sub-state-constituent-power> accessed 3 October 2023.

16 We return to this issue in s 3.

17 This is not to say that these alternatives cannot be interpreted in other ways, eg as means to merely increase political pressure on the UK government.

18 Scottish Parliament (n 1) 18.

19 See Alistair Grant, ‘Nicola Sturgeon Refutes Tory Claims She Is Planning “Wildcat” Referendum’ *The Herald* (4 May 2021) <www.heraldscotland.com/news/19278971.nicola-sturgeon-refutes-tory-claims-planning-wildcat-referendum/> accessed 16 June 2023.

20 Joe Watts, ‘SNP Refuse to Rule out Unofficial Scottish Independence Referendum in Defiance of Theresa May’ *The Independent* (17 March 2017) <www.independent.co.uk/news/uk/politics/snp-scottish-independence-referendum-theresa-may-uk-government-nicola-sturgeon-a7634586.html> accessed 16 June 2023.

21 See eg Scottish Sovereignty Research Group, ‘Routes to Independence’ <https://web.archive.org/web/20230327234500/https://scottishsovereigntyresearchgroup.org/routes-to-independence/> accessed 16 June 2023. See also ALBA, ‘ALBA Manifesto 2022’ (2022) 5 <www.albaparty.org/alba_manifesto_2022> accessed 16 June 2023; ALBA, ‘ALBA Manifesto 2021’ (2021) 3 <https://electricscotland.com/independence/manifestos/ALBA_Party_Manifesto2021.pdf> accessed 16 June 2023.

22 For an overview of different accounts of constituent power in Britain, see Joel Colón-Ríos, ‘Five Conceptions of Constituent Power’ (2014) 130 LQR 306; Alan Greene, ‘Parliamentary Sovereignty and the Locus of Constituent Power in the United Kingdom’ (2020) 18 ICON 1166, 1183–6. According to Loughlin, the concept of constituent power has English origins: Martin Loughlin, ‘Constituent Power Subverted: From English Constitutional Argument to British Constitutional Practice’ in Martin Loughlin and Neil Walker (eds), *The Paradox of Constitutionalism: Constituent Power and Constitutional Form* (OUP 2008).

23 Emmanuel Joseph Sieyès, *What Is the Third Estate?* (Michael Sonenscher ed, Hackett Publishing 2003) 136.

24 In contrast to widespread belief, however, Sieyès himself did not think that constituent power is completely unlimited, see Raffael N Fasel, ‘Constraining Constituent Conventions: Emmanuel Joseph Sieyès and the Limits of *Pouvoir Constituant*’ (2022) 20 ICON 1103.

25 Bruce Ackerman, *We the People: Foundations*, vol 1 (Belknap Press 1991) 6.

26 McHarg, ‘The Independence Referendum’ (n 8) 228.

27 ibid.

28 This is in line with Ackerman’s understanding, according to which even a period of an entire decade can count as a ‘constitutional moment’: see Daniel Young, ‘How Do You Measure a Constitutional Moment? Using Algorithmic Topic Modeling to Evaluate Bruce Ackerman’s Theory of Constitutional Change’ (2013) 122 Yale LJ 1990, 1998.

29 See eg McHarg, ‘The Independence Referendum’ (n 8) 247–8. See also Peter Niesen, ‘Two Cheers for Lost Sovereignty Referendums: Campaigns for Independence and the *Pouvoir Constituant Mixte*’ (2022) 23 German Law Journal 44, 54. See also Scottish government publications in the *Building a New Scotland* series, eg Scottish Government, ‘Renewing Democracy through Independence’ (2022), <www.gov.scot/publications/renewing-democracy-through-independence/> accessed 16 June 2023, which envisages independence as a precursor to enacting a written constitution.

30 Joel Colón-Ríos and others, ‘Constituent Power and Its Institutions’ (2021) 20 Contemporary Political Theory 926, 935. This is not to say that referenda are, on their own, ideal ways of expressing constituent power. See generally Andrew Arato, *Post Sovereign Constitution Making: Learning and Legitimacy* (OUP 2016).

31 Richard Tuck, *The Sleeping Sovereign: The Invention of Modern Democracy* (CUP 2015) 143, 167–8. For a different reading, see Rubinelli (n 9) 57.

32 Schmitt (n 9) 131.

33 ibid 132–3.

34 Sarbani Sen, *The Constitution of India: Popular Sovereignty and Democratic Transformations* (OUP 2011) 90, 95.

35 Colón-Ríos, *Constituent Power and the Law* (n 9) 264. For a similar distinction, see Stephen Tierney, *Constitutional Referendums: The Theory and Practice of Republican Deliberation* (OUP 2012) 11–12.

36 Colón-Ríos and others, ‘Constituent Power and Its Institutions’ (n 30) 938.

37 There can also be referenda on non-constitutional issues, as illustrated for example by Localism Act 2011, sch 2 para 1.

38 See ‘So-Called Referenda in Russian-Controlled Ukraine “Cannot Be Regarded as Legal”: UN Political Affairs Chief’ (*UN News*, 27 September 2022) <https://news.un.org/en/story/2022/09/1128161> accessed 16 June 2023.

39 There is some debate as to whether there is a constitutional convention that a referendum is required on a ‘fundamental constitutional issue’ such as secession: see eg House of Lords Select Committee on the Constitution, ‘Referendums in the United Kingdom, 12th Report of Session 2009–10’ (2010) para 94. Indeed, it has been said that since the fall of the USSR, referenda are ‘something *approaching* an international norm’ as a precursor to secession: Matt Qvortrup, ‘Referendums on Independence, 1860-2011’ (2014) 85 The Political Quarterly 57, 60 (emphasis added). However, in our view, neither an international norm nor a domestic constitutional convention have categorically been created.

40 See Stein Rokkan and Derek W Urwin, ‘Introduction: Centres and Periperhies in Western Europe’ in Stein Rokkan and Derek W Urwin (eds), *The Politics of Territorial Identity: Studies in European Regionalism* (Sage 1982) 11.

41 See for an overview Markus Patberg, ‘Challenging the Masters of the Treaties: Emerging Narratives of Constituent Power in the European Union’ (2018) 7 GlobCon 263.

42 See also Olivier Beaud, ‘The Founding Constitution: Reflections on the Constitution of a Federation and Its Peculiarity’ (2017) 17 Jus Politicum 33, 51.

43 Tierney, ‘We the Peoples’ (n 8) 230. See also Richard Rose, *Governing Without Consensus: An Irish Perspective* (Beacon Press 1971) 44.

44 Tierney, ‘We the Peoples’ (n 8) 232.

45 ibid (emphasis in original).

46 ibid.

47 Tierney uses the term ‘constitutional constituent authority’ to describe how sub-state nations’ constituent power is transformed in federal constitutions. However, the (revolutionary) exercise of constituent power to create a new constitution remains available to them: Stephen Tierney, *The Federal Contract: A Constitutional Theory of Federalism* (OUP 2022) 145–8.

48 For an overview, see Gavin Anderson and others, ‘The Independence Referendum, Legality and the Contested Constitution: Widening the Debate’ (*UK Constitutional Law Association Blog*, 31 January 2012) <https://ukconstitutionallaw.org/2012/01/31/gavin-anderson-et-al-the-independence-referendum-legality-and-the-contested-constitution-widening-the-debate/> accessed 16 June 2023.

49 Derek W Urwin, ‘Territorial Structures and Political Developments in the United Kingdom’ in Rokkan and Urwin, *The Politics of Territorial Identity* (n 40) 27.

50 James Mitchell, *Devolution in the UK* (Manchester UP 2009) 9.

51 For a different view, see Greene (n 22) 1171.

52 Aileen McHarg, ‘Devolution in Scotland’ in Jeffrey Jowell and Colm O’Cinneide (eds), *The Changing Constitution* (9th edn, OUP 2019) 278.

53 Aileen McHarg, ‘Unity and Diversity in the United Kingdom’s Territorial Constitution’ in Mark Elliott, Jason Varuhas, and Shona Wilson Stark (eds), *The Unity of Public Law?: Doctrinal, Theoretical and Comparative Perspectives* (Hart Publishing 2018) 280.

54 McHarg, ‘The Independence Referendum’ (n 8) 237.

55 ibid 242. See also David Torrance, *The Battle for Britain: Scotland and the Independence Referendum* (Biteback Publishing 2013) 18.

56 David Torrance, ‘Scottish Independence Referendum: Legal Issues’ (2022) 15 <https://researchbriefings.files.parliament.uk/documents/CBP-9104/CBP-9104.pdf> accessed 16 June 2023.

57 Canadian Supreme Court, *Reference re Secession of Quebec* [1998] 2 RCS 217, [134]–[135]. On internal self-determination, see in more detail Gudmundur Alfredsson, ‘The Right of Self-Determination in International Law’ in Olgun Akbulut and Elçin Aktoprak (eds), *Minority Self-Government in Europe and the Middle East* (Brill 2019) 17–28.

58 Jane A Hofbauer, *Sovereignty in the Exercise of the Right to Self-Determination* (Brill Nijhoff 2017) 70–1.

59 According to some, the UK may not only be a union state, but a (quasi-)federal state: see Anderson and others (n 48). See also Baroness Hale of Richmond, ‘The Supreme Court in the UK Constitution’ [2012] Legal Wales 23. Whether this is true need not concern us here because the finding that the UK is at least a union state is sufficient for viewing the Scottish people as a potential bearer of constituent power.

60 Niesen (n 29) 53. We are grateful to Mattias Kumm for this point. It could be argued that the Scottish Constitutional Convention’s role in establishing devolution was an exercise of constituent power, depending on how one defines ‘the people’ for the invocation of such a power: see Christine Bell, ‘Constitutional Transitions: The Peculiarities of the British Constitution and the Politics of Comparison’ [2014] PL 446, 460. The more important point for present purposes is that, in our view, constituent power *exists* to be used for such a purpose, even if there might be disagreement as to whether *this* was an example of its use.

61 See above n 8. According to Petkar, the UK Supreme Court implicitly acknowledged as much when recognising the democratic authority of a Scottish referendum: Pravar Petkar, ‘A Second Scottish Independence Referendum’ (n 15).

62 See also Niesen (n 29) 50–1 (arguing that an independence referendum such as the Scottish one may not be aimed primarily at secession but at achieving ‘*pouvoir constituant mixte*’—a form of dual sovereignty shared between the British people and the four nations). On the concept of *pouvoir constituant mixte*, see also Jürgen Habermas, ‘Citizen and State Equality in a Supranational Political Community: Degressive Proportionality and the *Pouvoir Constituant Mixte*’ (2017) 55 Journal of Common Market Studies 171; Markus Patberg, *Constituent Power in the European Union* (OUP 2021).

63 Tierney, ‘We the Peoples’ (n 8) 236.

64 See also Nico Krisch, ‘The Spanish Constitutional Crisis: Law, Legitimacy and Popular Sovereignty in Question’ (*Verfassungsblog*, 7 October 2017) <https://verfassungsblog.de/the-spanish-constitutional-crisis-law-legitimacy-and-popular-sovereignty-in-question/> accessed 16 June 2023. cf Tierney, ‘We the Peoples’ (n 8) 242, 244.

65
*Reference by the Lord Advocate* (n 3).

66 Scottish Parliament (n 1) 18.

67 Scottish Sovereignty Research Group, ‘Plebiscitary Election’ 1 <https://web.archive.org/web/20221015091555/https://ssrg.s3.eu-west-1.amazonaws.com/Plebiscitory+Election.pdf> accessed 16 June 2023.

68 ibid.

69 For an exception, see Marc Weller, ‘Forcing a Referendum on Scottish Independence?’ (*Lauterpacht Centre for International Law Blog*, 25 July 2022) <www.lcil.cam.ac.uk/blog/could-scotland-stage-independence-referendum-without-uk-approval-what-law-says-prof-marc-weller> accessed 16 June 2023. The idea of using an election as a plebiscite on independence also has precedent in Catalonia, as we will discuss in s 5.

70 Petkar, ‘Consultative Referendums’ (n 8) (emphasis in original).

71 McCorkindale and McHarg (n 6).

72 See above n 26.

73 Sieyès (n 23) 143.

74 ibid 136.

75 Schmitt (n 9) 131.

76 Yaniv Roznai, *Unconstitutional Constitutional Amendments: The Limits of Amendment Powers* (OUP 2017) 109. For the relational approach, which is critical of the dominant approach in this regard, see Hans Lindahl, ‘Constituent Power and Reflexive Identity: Towards an Ontology of Collective Selfhood’ in Loughlin and Walker (n 22); Martin Loughlin, ‘On Constituent Power’ in Michael W Dowdle and Michael A Wilkinson (eds), *Constitutionalism beyond Liberalism* (CUP 2017) 165–70. For a procedurally more demanding approach, see Arato, *Post Sovereign Constitution Making* (n 30).

77 We leave open the question of whether the elected representatives, once they move to drafting the new constitution, would directly exercise the people’s constituent power or whether they would exercise a limited, delegated power: on the distinction, see William Partlett, ‘The American Tradition of Constituent Power’ (2017) 15 ICON 955. For the related distinction between original and derived constituent power, see Roznai (n 76) 113–20; Colón-Ríos, *Constituent Power and the Law* (n 9) 13–17.

78 The Supreme Court emphasised that the ‘clarity’ provided by the ‘official and formal’ character of a referendum was what distinguished it from a mere ‘exercise in public consultation or a survey of public opinion’: *Reference by the Lord Advocate* (n 3) [78].

79 For a different view see Niesen (n 29) 54.

80 Kathleen Nutt, ‘Revealed: Wording Agreed for SNP Conference Motion on Plan B for Independence’ *The National* (26 September 2020) <www.thenational.scot/news/18750092.snp-conference-motion-plan-b-scottish-independence-wording-agreed/> accessed 16 June 2023.

81 Peter Davidson, ‘Nicola Sturgeon Speech in Full as First Minister Reacts to Supreme Court Verdict’ *Daily Record* (23 November 2022) <www.dailyrecord.co.uk/news/politics/nicola-sturgeon-speech-full-first-28564428> accessed 3 October 2023.

82 For example, in an April 2023 YouGov survey, in answer to the question ‘Should Scotland be an independent country?’, 44% of those aged 16–24 answered ‘yes’ compared to only 24% of those aged 65+: YouGov, Survey Results (17–20 April 2023) <https://docs.cdn.yougov.com/v27gr3cf2w/Results_230420_Scotland_VI&Others_W_.pdf> accessed 16 June 2023.

83 Glenn Campbell, ‘Are Elections the Way to Break the Indyref 2 Deadlock?’ (BBC News, 16 January 2023) <www.bbc.co.uk/news/uk-scotland-scotland-politics-64282309> accessed 16 June 2023.

84 Marc Weller, ‘Could Scotland Stage an Independence Referendum without UK Approval? What the Law Says’ (*Lauterpacht Centre for International Law Blog*, 18 February 2021) <www.lcil.cam.ac.uk/blog/could-scotland-stage-independence-referendum-without-uk-approval-what-law-says-prof-marc-weller> accessed 16 June 2023.

85 On Catalonia, where this was an issue, see Jaume López and Marc Sanjaume-Calvet, ‘The Political Use of de Facto Referendums of Independence: The Case of Catalonia’ (2020) 56 Representation 501, 507.

86 Each voter casts two votes in elections to the Scottish Parliament: one for their constituency MSP and the second for a political party. The second vote is then used to elect ‘list’ MSPs so that the overall number of a party’s MSPs is more in proportion to their share of the vote than under a first-past-the-post system.

87 See also Sebastian Balfour, ‘Catalonia Is Facing a Deeply Uncertain Future—Whether Inside or Outside of Spain’ (*LSE European Policy and Politics Blog*, 6 October 2015) <https://blogs.lse.ac.uk/europpblog/2015/10/06/catalonia-is-facing-a-deeply-uncertain-future-whether-inside-or-outside-of-spain/> accessed 16 June 2023 (discussing the Catalan plebiscitary election of 27 September 2015).

88 Petkar, ‘Consultative Referendums’ (n 8).

89 For a more sceptical take, see Elisenda Casanas Adam, ‘Alternative Pathways to an Independence Referendum: Some Reflections Based on the Experience of Catalonia’ (2021) 25 Edinburgh Law Review 125, 127.

90 See Vernon Bogdanor, ‘General Election: Do Party Manifestos Still Matter?’ *Financial Times* (15 April 2015). See also BMG Research, ‘BMG Research Poll: Two-thirds of People Don’t Read Political Manifestos’ (2017) <www.bmgresearch.co.uk/bmg-research-poll-10-people-dont-know-manifesto/> accessed 16 June 2023.

91 Such as the UK Parliament constituency of Banff and Buchan, held by former First Minister Alex Salmond from 1987 to 2010, but currently held by the Scottish Conservatives.

92 See López and Sanjaume-Calvet (n 85) 513.

93 YouGov, ‘Survey Results: “Would You Support or Oppose Making It Compulsory for People to Vote in General Elections?”’ (2022) <https://docs.cdn.yougov.com/p8b9cqxrku/YouGov%20-%20Compulsory%20Voting%20Results.pdf> accessed 16 June 2023.

94 This is not to say, however, that referenda are paragons of clarity: see Tierney, *Constitutional Referendums* (n 35) 17.

95 Scottish Parliament (n 1) 14.

96 Sieyès (n 23) 135.

97
*Reference by the Lord Advocate* (n 3) [78].

98 Mattias Kumm, ‘Constituent Power, Cosmopolitan Constitutionalism, and Post-Positivist Law’ (2016) 14 ICON 697, 701.

99 Tierney, ‘We the Peoples’ (n 8) 240.

100 Of course, constituent power in the context of Indyref 2 differs from that of the French Revolution in that the former would not occur in a unitary state. However, in our view, the UK’s character as a union state does not fundamentally change the equation. If one assumes, as we do, that Scotland is a constituent part of the UK and has not lost its own constituent power, any potential division of power within the UK along the lines of *pouvoir constituant mixte* or similar notions does not affect Scotland’s revolutionary power to withdraw without authorisation. See also above n 47.

101
*Reference by the Lord Advocate* (n 3) [78].

102 See Mikołaj Barczentewicz, ‘Constituent Power and Constituent Authority’ (2021) 52 Conn L Rev 1313, 1329.

103 Alternatively, one might also argue that they lack constituent authority: Richard S Kay, ‘Constituent Authority’ (2011) 59 Am J Comp L 715. We are grateful to Peter Niesen for this point.

104 This focus is especially warranted if one follows, as we do here, the dominant view in international law, according to which recognition of states merely has declaratory, not constitutive effect: see Patrick CR Terry, ‘The Recognition of New States in Times of Secession: Is State Recognition Turning into Another Means of Intervention?’ in Kevin YL Tan (ed), *Asian Yearbook of International Law*, vol 20 (Brill 2014) 57–8.

105 McHarg, ‘The Independence Referendum’ (n 8) 228. See also Scottish Parliament (n 1) 14.

106 Andrew Learmonth, ‘Over Two-Thirds of Scots Want to Rejoin the EU’ *The Herald* (22 August 2022) <www.heraldscotland.com/politics/20681698.two-thirds-scots-want-rejoin-eu/> accessed 16 June 2023.

107 SNP, ‘SNP Manifesto—General Election 2019’ (2019) 10 <www.snp.org/general-election-2019/> accessed 16 June 2023. See also Scottish Government, ‘Scotland’s Right to Choose: Putting Scotland’s Future in Scotland’s Hands’ (2019) 20; SNP, ‘SNP 2021 Manifesto: Scotland’s Future, Scotland’s Choice’ (2021) 12 <www.snp.org/manifesto/> accessed 16 June 2023.

108 UN Charter, art 2(4); Hermann-Josef Blanke and Yasser Abdelrehim, ‘Catalonia’s Independence—Is There a Way in International and European Union Law?’ in Klaus-Jürgen Nagel and Stephan Rixen (eds), *Catalonia in Spain and Europe: Is There a Way to Independence?* (Nomos 2015) 63.

109 See Stefan Oeter, ‘The Role of Recognition and Non-recognition with Regard to Secession’ in Christian Walter, Antje von Ungern-Sternberg, and Kavus Abushov (eds), *Self-Determination and Secession in International Law* (OUP 2014) 53. See also UN General Assembly Resolution 1514 (XV) 14 December 1960.

110 The ICJ left the question open as to whether there is such a right and noted the existence of different views on this question: Accordance with International Law of the Unilateral Declaration of Independence in Respect of Kosovo, Advisory Opinion, ICJ Reports 2010, 403, [82]. But see also the separate opinion of Judge Yusuf, [11].

111 Oeter (n 109) 57.

112
*Reference re Secession of Quebec* (n 57) [134]–[135].

113 See eg Krisch (n 64).

114
*Reference by the Lord Advocate* (n 3).

115 Advisory Opinion (n 110) [49].

116 ibid [84].

117 Marc Weller, ‘The Sounds of Silence: Making Sense of the Supposed Gaps in the Kosovo Opinion’ in Marko Milanovic and Michael Wood (eds), *The Law and Politics of the Kosovo Advisory Opinion* (OUP 2015) 195.

118 Advisory Opinion (n 110) [80].

119 Weller, ‘The Sounds of Silence’ (n 117) 198.

120 Which are, according to art 1 of the Montevideo Convention on Rights and Duties of States 1933: (i) ‘permanent population’; (ii) ‘defined territory’; (iii) ‘government’; and (iv) ‘capacity to enter into relations with other states’; see further Terry (n 104) 64–73.

121 Weller, ‘The Sounds of Silence’ (n 117) 198; see also Terry (n 104) 85; Oeter (n 109) 51. On what counts as a prohibited form of external intervention, see Philip Kunig, ‘Intervention, Prohibition Of’, *Max Planck Encyclopedia of Public International Law* (2008) <https://opil.ouplaw.com/view/10.1093/law:epil/9780199231690/law-9780199231690-e1434?prd=EPIL> accessed 16 June 2023.

122 See also McCorkindale and McHarg (n 6). The orthodox view in international law is that recognition of statehood is a political, not a legal act, see eg Blanke and Abdelrehim (n 108) 79. See also above n 104.

123
*Reference by the Lord Advocate* (n 3) [88].

124 ibid.

125 For a useful overview of the history of Catalonia’s struggle for independence, see Blanke and Abdelrehim (n 108) 56–62.

126 Junts pel Sí, ‘Programa electoral’ (2015) 32 <www.esquerra.cat/partit/programes/c2015_programa.pdf> accessed 16 June 2023.

127 The 125-page manifesto, although focused on independence, also touched on many additional issues, such as the political, welfare, economic, and cultural institutions of the new state: ibid.

128 Balfour (n 87).

129 Casanas Adam, ‘Alternative Pathways’ (n 89) 128.

130 ibid.

131 López and Sanjaume-Calvet (n 85) 507; Constitutional Court of Spain, 114-2017, 17 October 2017.

132 López and Sanjaume-Calvet (n 85).

133 ibid 507–8.

134 ibid 505.

135 ibid.

136 Maurits Helmich, ‘Spain, Catalonia, and the Supposed Authority of the Judiciary: On the Self-Constitutive Moment in Adjudication’ (2020) 2 Jus Cogens 259, 260. The Spanish President Pedro Sánchez recently pardoned the nine leaders: Mark Nayler, ‘Spain’s Prime Minister Can’t Win When It Comes to Catalonia’ (*Foreign Policy*, 9 July 2021) <https://foreignpolicy.com/2021/07/09/spain-catalonia-independence-pardons-prime-minister-pedro-sanchez/> accessed 16 June 2023.

137 European Commission, ‘Statement on the Events in Catalonia’ (2017) <https://ec.europa.eu/commission/presscorner/detail/en/STATEMENT_17_3626> accessed 16 June 2023.

138 Paula Ajumobi, ‘Catalonia: Potential Sovereignty in the Era of Controversial Self-Determination’ (2021) 53 NYU Journal of International Law and Politics 63, 71.

139 See Antoni Abat i Ninet, ‘The Spanish Constitution, the Constitutional Court, and the Catalan Referendum’ in Nagel and Rixen (n 108); Weller, ‘Forcing a Referendum’ (n 69). See also Marc Weller, ‘The UK Supreme Court Reference on a Referendum for Scotland and the Right to Constitutional Self-Determination: Part II’ (*EJIL: Talk!*, 13 December 2022) <www.ejiltalk.org/the-uk-supreme-court-reference-on-a-referendum-for-scotland-and-the-right-to-constitutional-self-determination-part-ii/> accessed 16 June 2023.

140 See eg Andrea Romano, ‘Constituent Power and Independence Processes: Problems and Perspectives in the Light of the Catalan Experience’ (2018) 27 Revista d’Estudis Autonòmics i Federals 41, 73. See, similarly, Krisch (n 64); Barczentewicz (n 102) 1328–9. Even prior to the 2017 referendum, authors such as Edgar Illas argued that the Catalans were the bearer of constituent power: Edgar Illas, ‘Is Catalan Separatism a Progressive Cause?’ (2014) 10 Hispanic Journal of Theory and Criticism 11; see also Edgar Illas, ‘Catalan Secessionism and Globalization’ (2021) 22 Georgetown Journal of International Affairs 213, 213. On Quebec, see eg Tierney, *Constitutional Referendums* (n 35) 144–5; Jonathon W Penney, ‘Deciding in the Heat of the Constitutional Moment: Constitutional Meaning and Change in the Quebec Secession Reference’ (2005) 28 Dalhousie Law Journal 45, 220. For more sceptical accounts, see eg Niesen (n 29) 53; José Luis Martí, ‘Seven Steps to Hell: The Catalan Conflict in Full Escalation Mode’ (*Verfassungsblog*, 25 October 2017) <https://verfassungsblog.de/seven-steps-to-hell-the-catalan-conflict-in-full-escalation-mode/> accessed 16 June 2023.

141 Weller, ‘Forcing a Referendum’ (n 69).

142 ibid.

143 See Blanke and Abdelrehim (n 108) 73.

144 ibid 80.

145 Martí (n 140).

146 Guy Hedgecoe, ‘The Real Loser of the Catalan Secession Push: Catalan Nationalists’ *Politico* (30 September 2022) <www.politico.eu/article/catalanonia-secession-spain-nationalist-vote/> accessed 4 January 2023.

147 See Casanas Adam, ‘Alternative Pathways’ (n 89) (focusing on plebiscitary elections).

148 Tickell (n 5) 85.

149 On the need and indeed obligation to negotiate in a democratic society, see *Reference re Secession of Quebec* (n 57) [93]–[97]. See also Casanas Adam, ‘Alternative Pathways’ (n 89) 129. In passing, we lament that the UK Supreme Court in *Reference by the Lord Advocate* (n 3) was not as bold as the Canadian Supreme Court in emphasising the importance of political negotiation as a way to break the current impasse. The UK Supreme Court’s approach is consistent with McHarg’s observation in the context of *R (on the application of Miller and another) v Secretary of State for Exiting the European Union* [2017] UKSC 5 that the justices have chosen to view themselves as the ‘defenders of the rule of law, rather than as guardians of the constitution’: Aileen McHarg, ‘Constitutional Change and Territorial Consent: The *Miller* Case and the Sewel Convention’ in Mark Elliott, Jack Williams, and Alison L Young (eds), *The UK Constitution after* Miller*: Brexit and Beyond* (Hart Publishing 2018) 179.

150 Stephen Tierney, ‘A Second Scottish Independence Referendum without a s 30 Order? A Legal Question That Demands a Political Answer’ (Centre on Constitutional Change, 21 March 2017) <www.centreonconstitutionalchange.ac.uk/opinions/second-scottish-independence-referendum-without-s30-order-legal-question-demands-political> accessed 16 June 2023.

151 See BBC News, ‘What Does “Once in a Generation” Mean?’ (16 January 2020) <www.bbc.co.uk/news/uk-scotland-scotland-politics-51120175> accessed 4 January 2023.

152 SNP, ‘Application to Intervene in the Reference by the Lord Advocate under Paragraph 34 of Schedule 6 of the Scotland Act 1998 in Relation to Whether the Question for a Referendum on Scottish Independence Contained in the Proposed Bill Relates to Reserved Matters’, [20].

153 Aileen McHarg, ‘Unity and Diversity in the United Kingdom’s Territorial Constitution’ in Elliott, Varuhas, and Stark (n 53) 297–9.

154 Scottish Government, ‘Human Rights’ <www.gov.scot/policies/human-rights/> accessed 16 June 2023. See also BBC News, ‘Nicola Sturgeon Says Gender Reform Row Will Go to Court’ (18 January 2023) <www.bbc.co.uk/news/uk-scotland-scotland-politics-64264063> accessed 16 June 2023.

155 Tickell (n 5) 85. See HC Deb 14 December 2022, vol 724, cols 1176–8 (Pete Wishart MP).

156 YouGov, Survey Results (n 82).

157 That is to say, a referendum facilitated by legislation passed pursuant to a s 30 Order, as per Indyref 1: Reuters, ‘Scottish Government Publishes Draft Bill for Second Independence Referendum’ (20 October 2016) <www.reuters.com/article/us-britain-eu-scotland-idUSKCN12J2S0> accessed 16 June 2023.

